# Impact of charging infrastructure construction on electric vehicle diffusion based on a multi-agent model

**DOI:** 10.1016/j.isci.2025.112257

**Published:** 2025-03-20

**Authors:** Yingying Zheng, Donghui Liu, Feng An, Jian Wang, Xiangyun Gao, Nanfei Jia

**Affiliations:** 1 School of Management, Shijiazhuang Tiedao University, Shijiazhuang 050043, China; 2 Research Institute of Engineering Management, Shijiazhuang Tiedao University, Shijiazhuang 050043, China; 3 School of Economics and Management, Beijing University of Chemical Technology, Beijing 100029, China; 4 School of Economics and Law, Shijiazhuang Tiedao University, Shijiazhuang 050043, China; 5 School of Economics and Management, China University of Geosciences, Beijing 100083, China; 6 Key Laboratory of Carrying Capacity Assessment for Resource and Environment, Ministry of Natural Resources of the People’s Republic of China, Beijing 100083, China; 7 School of Computer and Artificial Intelligence, Beijing Technology and Business University, Beijing 102488, China

**Keywords:** Electrochemical energy storage, Energy resources, Energy Modeling

## Abstract

To explore the impact of charging infrastructure on electric vehicles (EVs) diffusion, a multi-agent model of EVs-charging infrastructure construction (EV-CIC) is established based on complex adaptive system (CAS). The simulation examines four infrastructure factors and two policy interventions. The results show that the installation rate of private charging piles has the greatest impact, increasing market share by 4% for every 10% rise, followed by the number of public charging piles with 1.58% per 100 units, failure rate with 1.3% per 10% reduction, and charging price with 1% per 10 yuan. High subsidy rates show strong effects in the early stages, while sharing policies for private charging piles show better long-term benefits, increasing market share by 13% compared to non-sharing scenarios. In conclusion, private charging piles, whether through increasing installation rates or enhancing sharing policies, could lead to significant breakthroughs in promoting the development of the EV market.

## Highlights

• Impact of CIC on EV market is assessed considering multi-agents and various factors
• The interactions between agents are analyzed in a complex adaptive system
• Dynamics in the EV market are developed based on a multi-agent model of EV-CIC
• Private charging infrastructure has a significant impact on EV market penetration

## Graphical abstract

**Figure:**
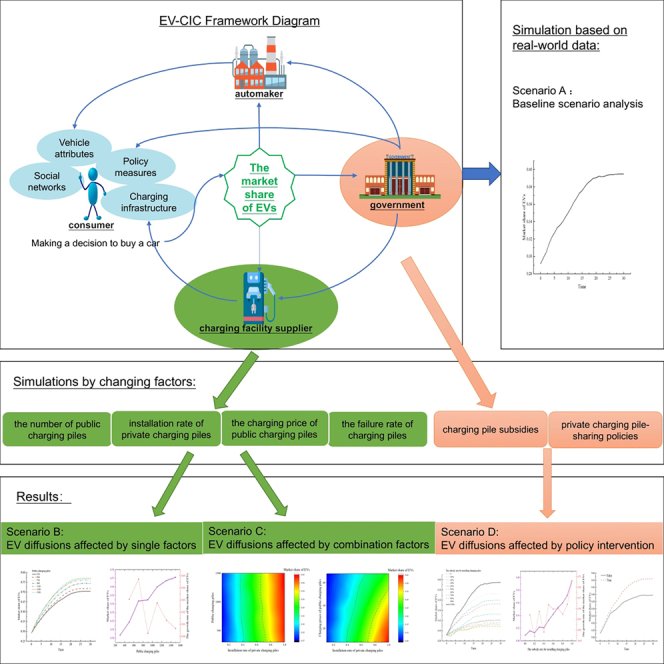

## Introduction

Electric vehicles (EVs) are seen as a breakthrough for a new round of economic growth and a fundamental way to achieve transportation energy transformation.^1^ Due to the advantages of EVs such as zero tailpipe emissions and low energy consumption, many countries are actively working to increase the market share of EVs. However, EVs have not yet been accepted by the public because users have range anxiety. Specifically, it is difficult for users to find charging piles, especially when the EV cannot reach the destination.^2^ This is because the number of charging facilities directly affects the convenience of users and is an important factor affecting the popularity of EVs.^3^ The current charging infrastructure is not perfect, although charging facilities have advantages such as a high level of intelligent networking and eco-friendliness.^4^ As a result, it is imperative to promote the construction of charging infrastructure.

However, there are still many issues in the development of charging infrastructure, limiting the development of EVs. First, there is the issue of the number of charging facilities. For public charging piles, for example, China accounts for over 60% of the global EV market share, but there are only 2.726 million public charging posts in China by the end of 2023, representing a ratio of less than 1 station for every 5 EVs^5^ Moreover, the ratio of EVs to charging piles in the public charging field is unbalanced among regions, leading to regional differences in user’s recharge anxiety. For private charging piles, for example, the installation rate of private charging piles is still low in China. The number of private charging posts is 5.87 million, and only 37.82% of EV users installed private charging posts with their cars.^6^ Second, the charging speed and reliability of charging facilities have a more obvious effect on expanding the scale of EV adoption, compared with simply increasing the number of charging piles.^7^ In China, the number of direct current piles with public fast charging performance is only 1.14 million, and the pile-vehicle ratio is less than 1:13.^8^ And the current failure rate of public charging piles is close to one-third, which limits the improvement of the average charging speed of vehicles, and limits the user experience satisfaction and convenience of use.^8^ Dynamic pricing mechanisms reduce average charging time by promoting orderly charging behavior. However, this mechanism harms the user experience by forcing them to choose between higher charges during their preferred period and inconvenient charging times.^9^

Therefore, considering the current situation, charging infrastructure significantly impacts the development of EVs through its convenience, reliability, cost-effectiveness, intelligent networking, and eco-friendliness. However, there are still some challenges, such as an insufficient number of public charging piles, low installation of private charging piles, high failure rates, a low proportion of fast charging options, and unstable electricity prices. This study aims to discuss through quantitative analysis how addressing these issues could affect the diffusion of the EV market, thereby providing a theoretical basis for policy implication regarding the construction of EV charging infrastructure.

In fact, there has already been an increasing amount of research on the impact of charging infrastructure development on the market diffusion of EVs. Various methodologies have been employed in these studies, including econometric methods, game theory, operations research optimization, machine learning, system dynamics, and multi-agent modeling. Econometric methods (such as panel data analysis, discrete choice models, and structural equation modeling) have primarily been used to verify the causal relationship between charging facilities and EV market development, as well as to analyze consumer choice behavior and preference characteristics.^10^^,^^11^^,^^12^^,^^13^ Game theory is mainly used to study the competitive behavior of charging operators and the interaction mechanism between government and enterprises.^14^^,^^15^^,^^16^ Operations research optimization methods have been mainly applied to solve problems related to charging facility location, scale, and pricing.^17^^,^^18^^,^^19^^,^^20^ With the advancement of data availability and computational capabilities, machine learning methods have also been widely adopted, utilizing deep learning, reinforcement learning, and neural networks to predict charging demand and market development trends.^17^^,^^21^^,^^22^ Compared to these methods, system dynamics and multi-agent modeling offer superior capabilities for studying the complex systems of EV markets.^23^^,^^24^ Considering that the impact of charging infrastructure development on EV market diffusion is a complex systemic issue involving dynamic interactions among multiple stakeholders (such as consumers, automakers, and governments), and that there is significant heterogeneity within these entities (e.g., differences in consumers’ environmental awareness and purchasing preferences)^25^^,^^26^^,^^27^^,^^28^^,^^29^. Additionally, charging infrastructure has distinct spatial attributes, with its layout directly affecting consumer convenience and willingness to adopt EVs.^27^^,^^30^^,^^31^^,^^32^ In comparison to system dynamics, multi-agent modeling not only simulates the behavioral characteristics and interactions of multiple market entities but also better addresses individual heterogeneity.^26^^,^^29^ It can also effectively handle the spatial attributes of charging facilities, accurately reflecting the impact of facility layout on consumer convenience.^30^^,^^32^ Furthermore, multi-agent modeling demonstrates strong methodological integration capabilities, which can integrate methods such as consumer choice models into the modeling framework and support simulation analysis of various scenarios.^24^^,^^26^^,^^32^ Therefore, multi-agent modeling is selected as the core methodology to investigate the influence of charging facilities on the EV market.

The majority of multi-agent models that study the impact of charging infrastructure on EV market diffusion focus on modeling consumer agents. Huang et al. integrated consumer preferences, charging infrastructure, and government subsidies to depict consumer decision logic in ABM.^33^ Liu et al. developed a Bayesian optimization-based consumer demand simulation model in ABM, considering heterogeneous characteristics such as consumer income levels and environmental awareness.^32^ Xu et al. constructed a two-stage consumer choice model, where the word-of-mouth (WOM) effect and consumers’ social networks are also considered in the ABM.^27^ These studies generally consider factors such as consumer heterogeneity, social network influences, and technology acceptance to describe consumer purchase decision processes. Besides consumers, automakers and governments have also received extensive attention. Yu et al. constructed an evolutionary game model in ABM and found that the dual-credit policy has a significant promoting effect on traditional automakers’ technological innovation and electrification transition.^34^ Demartini et al. placed automakers within the entire supply chain context to study the impact of electric and net-zero economy transition on automotive supply chains and relevant stakeholders.^35^ Additionally, in some studies, automakers’ behavioral decisions were simplified, primarily focusing on consumer behavior and market diffusion.^27^^,^^33^ Regarding government agents, research primarily focuses on evaluating the effectiveness of various policy instruments and their combinations, including subsidy policies, orderly charging policies, carbon trading mechanisms, battery recycling regulations, and charging infrastructure initiatives, along with their synergistic effects.^32^^,^^36^^,^^37^^,^^38^ However, charging facility suppliers, as crucial market participants, are notably underrepresented in existing research. On one hand, current studies primarily concentrate on techno-economic analysis and spatial layout optimization of charging facilities. For instance, Li et al. focused on charging pile location problems using data-driven approaches in Sydney^39^; Zarazua et al. investigated charging business models^40^; and Chen et al. explored facility operational efficiency through evolutionary analysis.^16^ On the other hand, while some studies have begun to examine the role of charging facility suppliers within the system, these studies typically consider limited stakeholder interactions rather than systematic multi-agent analysis. For instance, Zhao et al. analyzed competitive charging pile deployment considering existing competitors^41^; and Sun et al. explored dynamic interactions among government, charging facility suppliers, and consumers.^42^ Therefore, there is an urgent need to construct an analytical framework that integrates consumers, automakers, government, and charging facility suppliers, to investigate the impact of charging infrastructure on EV market diffusion.

Furthermore, the dimension of charging infrastructure remains relatively limited, although existing research has begun to examine the impact of charging infrastructure on EV market diffusion. Specifically, existing literature primarily focuses on two aspects: spatial accessibility and pricing factors of charging services. Regarding spatial accessibility, Esmaili et al. investigated how charging network coverage influences consumer purchase intentions.^43^ Sun et al. analyzed the relationship between charging facility density and market penetration rate.^42^ Chen et al. explored the impact of facility layout on adoption decisions based on consumer travel characteristics.^16^ In terms of pricing factors, Wu et al. examined how charging costs affect consumer usage behavior.^44^ Yang et al. studied the interaction between pricing strategies and market diffusion.^45^ However, a comprehensive evaluation of charging infrastructure should extend beyond spatial accessibility and pricing to include convenience, intelligence level, environmental sustainability, and stability. Additionally, as crucial components of charging infrastructure, private charging pile construction and sharing policies may significantly influence consumer experience and purchase decisions. While some studies have begun to pay attention to these factors, such as Zhao et al. analyzing the effect of charging speed on consumer choice,^46^ and Emodi et al. exploring the role of smart reservation systems.^47^ These studies often consider features in isolation. There is a lack of systematic analysis of multiple charging facility characteristics. Therefore, an analytical framework integrating multiple dimensions of charging facility characteristics is urgently needed to study their impact on market diffusion.

Based on the previous analysis and complex adaptive systems (CASs) theory, this study establishes a multi-agent model incorporating consumers, automakers, government, and charging facility suppliers. The model comprehensively considers multiple aspects of charging infrastructure development along with relevant policy factors to analyze the impact of charging infrastructure on EV market diffusion. The innovation of this study can be summarized into three points. First, it considers multiple dimensions of charging infrastructure, such as the installation rate of private charging piles and policy interventions, including charging pile subsidies and private charging pile-sharing policies, besides the factors of the number of public charging piles, the failure rate of charging piles, and the charging price of public charging piles, thoroughly exploring their impact on the diffusion of the EV market. Second, considering the behavior of charging facility suppliers, a multi-agent model of EVs-charging infrastructure construction (EV-CIC) is constructed based on CAS, incorporating agents like charging facility suppliers, consumers, automakers, and the government. Third, our findings suggest that the installation rate of private charging piles has the most significant impact on market share. And the promotion of private charging piles, whether through increased installation rates or enhanced sharing policies, could lead to significant breakthroughs in promoting the development of the EV market. This study provides a comprehensive consideration of the adaptability and interactivity among multiple agents, exploring the multifaceted influence of charging infrastructure on the diffusion of the EV market. It not only enriches the development of CAS and the application of multi-agent modeling simulations, but provides practical recommendations and theoretical bases for policymakers and industry stakeholders.

## Results and discussion

### Baseline scenario analysis

**Figure 1:**
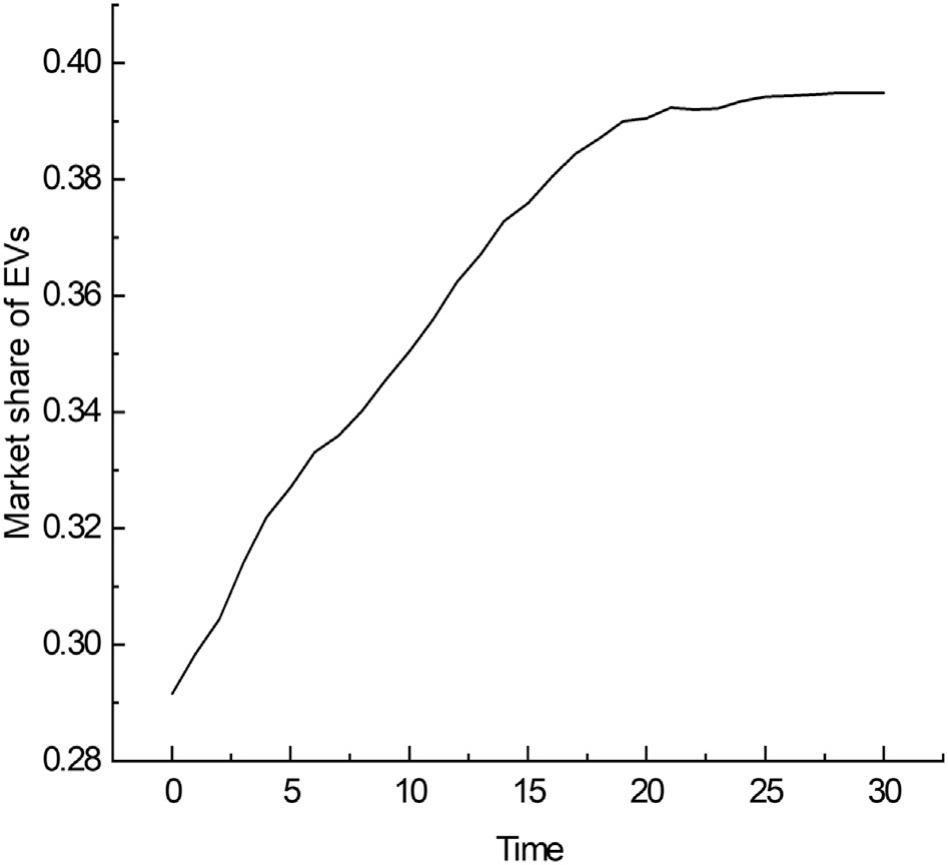
Market share of EVs in the baseline scenario

The baseline scenario is used as a reference to analyze the impact of factors on the market share of EVs. Figure 1 shows the evolutionary trend under the baseline scenario, with the initial parameters of 300 public charging piles, a failure rate of 30%, a private charging pile installation rate of 40%, and an installation subsidy rate of 100%. As time progressed, the market share of EVs grew steadily. After 20 ticks, the growth rate slowed and stabilized at approximately 40%. This process follows an S-shaped growth curve, aligning with the innovation diffusion curve proposed by Rogers and Simon.^48^ However, the market share of EVs remains below 50%.

### Scenario B analysis: Single factor changes in charging facility construction

#### Scenario B-1: Changes in the number of public charging piles

**Figure 2:**
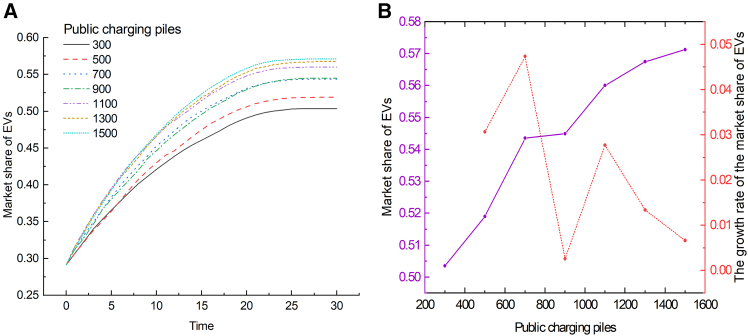
The impact of the number of public charging piles on the market share of EVs (A) Shows the evolution curves of the market share of EVs over time under varying numbers of public charging piles. (B) Shows the relationship between the number of public charging piles and the market share of EVs (solid purple line), and the growth rate of the market share of EVs (red dashed line), at Time = 30.

To investigate the impact of the number of public charging piles on the market share of EVs, simulations were conducted and the results are shown in Figure 2. Initially, the market share of EVs shows a continuous upward trend over time. Especially in the early stages (approximately the first 0–10 ticks), the market share of EVs in all scenarios increases rapidly, indicating that the initial adoption rate is strongly affected by the availability of charging infrastructure. In addition, the number of charging piles is positively correlated with the market share of EVs, confirming the key role of charging infrastructure construction in promoting the widespread adoption of EVs. However, the marginal benefit of increasing charging piles gradually decreases. From the perspective of market stability, 700 charging piles achieve the best utility under the setting of this model. Therefore, to maximize the adoption rate of EVs, policies should reasonably increase the number of public charging piles. In the simulation scenario, the number of 700 charging piles is a suitable choice.

#### Scenario B-2: Changes in the installation rate of private charging piles

**Figure 3:**
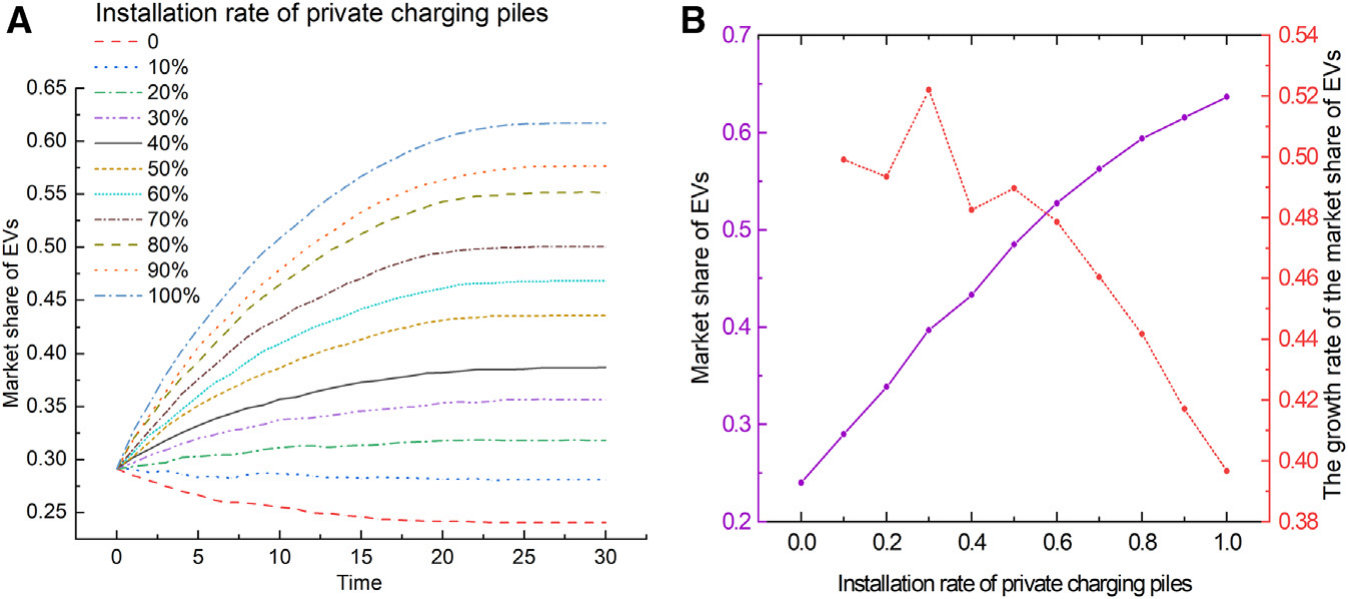
The impact of the installation rate of private charging piles on the market share of EVs (A) Shows the evolution curves of the market share of EVs over time under varying installation rates of private charging piles. (B) Shows the relationship between the installation rate of private charging piles and the market share of EVs (solid purple line), and the growth rate of the market share of EVs (red dashed line), at Time = 30.

To study the impact of private charging pile installation rate on EV market share, a simulation was conducted and the results are shown in Figure 3. When the installation rate is below 20%, the EV market share shows a downward trend. This suggests that initial efforts must focus on promoting the widespread adoption of private charging piles to promote the development of the EV market. The installation rate of private charging piles has a significant positive impact on the EV market share. As the installation rate of private charging piles increases, the market share grows steadily, which may be because private charging solutions are the key to alleviating consumers’ charging anxiety. The marginal growth rate ranges from 0.39 to 0.52 stably, indicating a steady marginal benefit in private charging pile installations. This stability likely stems from a sustained long-term demand for private charging facilities. This shows that increasing the installation rate of private charging piles is crucial to promoting the sustained and healthy development of the EV industry.

#### Scenario B-3: Changes in the failure rate of charging piles

**Figure 4:**
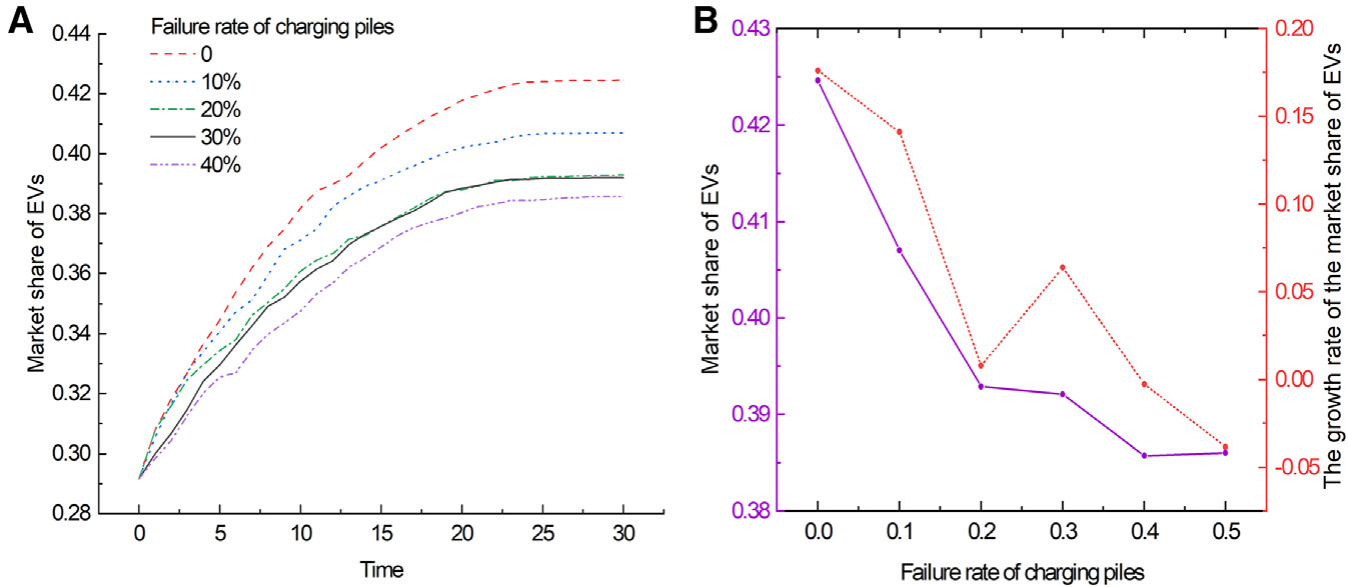
The impact of charging pile failure rate on the market share of EVs (A) Shows the evolution curves of the market share of EVs over time under varying failure rates of charging piles. (B) Shows the relationship between the failure rate of charging piles and the market share of EVs (solid purple line), and the growth rate of the market share of EVs (red dashed line), at Time = 30.

To study the effect of charging pile failure rate on the market share of EVs, simulations were conducted and the results are shown in Figure 4. The findings revealed an inverse relationship between charging pile failure rates and EV adoption, aligning with initial hypotheses. It is worth noting that a decrease in failure rate from 33% to 23% did not significantly increase the market share of EVs; however, a decrease to 13% showed a significant positive effect. In contrast, an increase from 33% to 43% had a significant negative effect on market share. These observations suggest a nonlinear relationship between charging pile failure rates and EV market share. At lower failure rates, market share changes appear less pronounced, indicating reduced user sensitivity to minor variations in failure rates. In this case, a significant reduction in the failure rate is required to significantly increase market share, while a slight increase in the failure rate will have an adverse impact on the EV market. Therefore, to promote the development of the EV market, it is crucial to reduce the failure rate of charging piles and improve their reliability and stability.

#### Scenario B-4: Changes in the charging prices of public charging piles

**Figure 5:**
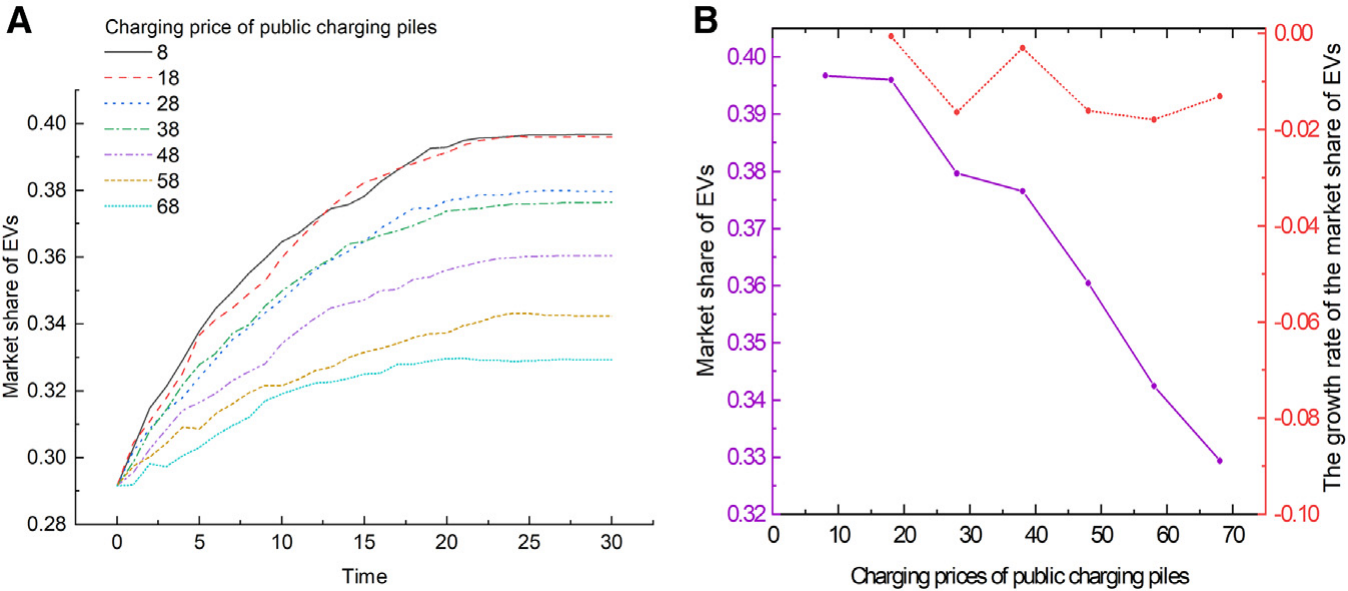
The impact of the charging price of public charging piles on the market share of EVs (A) Shows the evolution curves of the market share of EVs over time under varying charging prices of public charging piles. (B) Shows the relationship between the charging prices of public charging piles and the market share of EVs (solid purple line), and the growth rate of the market share of EVs (red dashed line), at Time = 30.

To study the impact of the charging prices of public charging pile on the market share of EVs, a simulation was conducted, and the results are shown in Figure 5. As the charging price increases from low to high, the overall market share shows a downward trend. Specifically, as the charging price increases from 8 yuan/100 km to 68 yuan/100 km, the market share gradually decreases. This trend reflects consumers’ price sensitivity regarding operational costs in their EV purchase decisions. Higher charging prices may reduce the attractiveness of EVs, particularly when considering the total cost of ownership over the vehicle’s life cycle. Therefore, maintaining stable charging prices is a key strategy to maintain the long-term development of the EV market. In particular, lower charging costs through policy support and technological progress can improve the market competitiveness and acceptance of EVs.

### Scenario C analysis: Changes in combination factors in charging facility construction

According to the above simulation results, under the steady state, the installation rate of private charging piles has the greatest impact, increasing market share by 4% for every 10% rise, followed by the number of public charging piles with 1.58% per 100 units, failure rate with 1.3% per 10% reduction, and charging price with 1% per 10 yuan.

**Figure 6:**
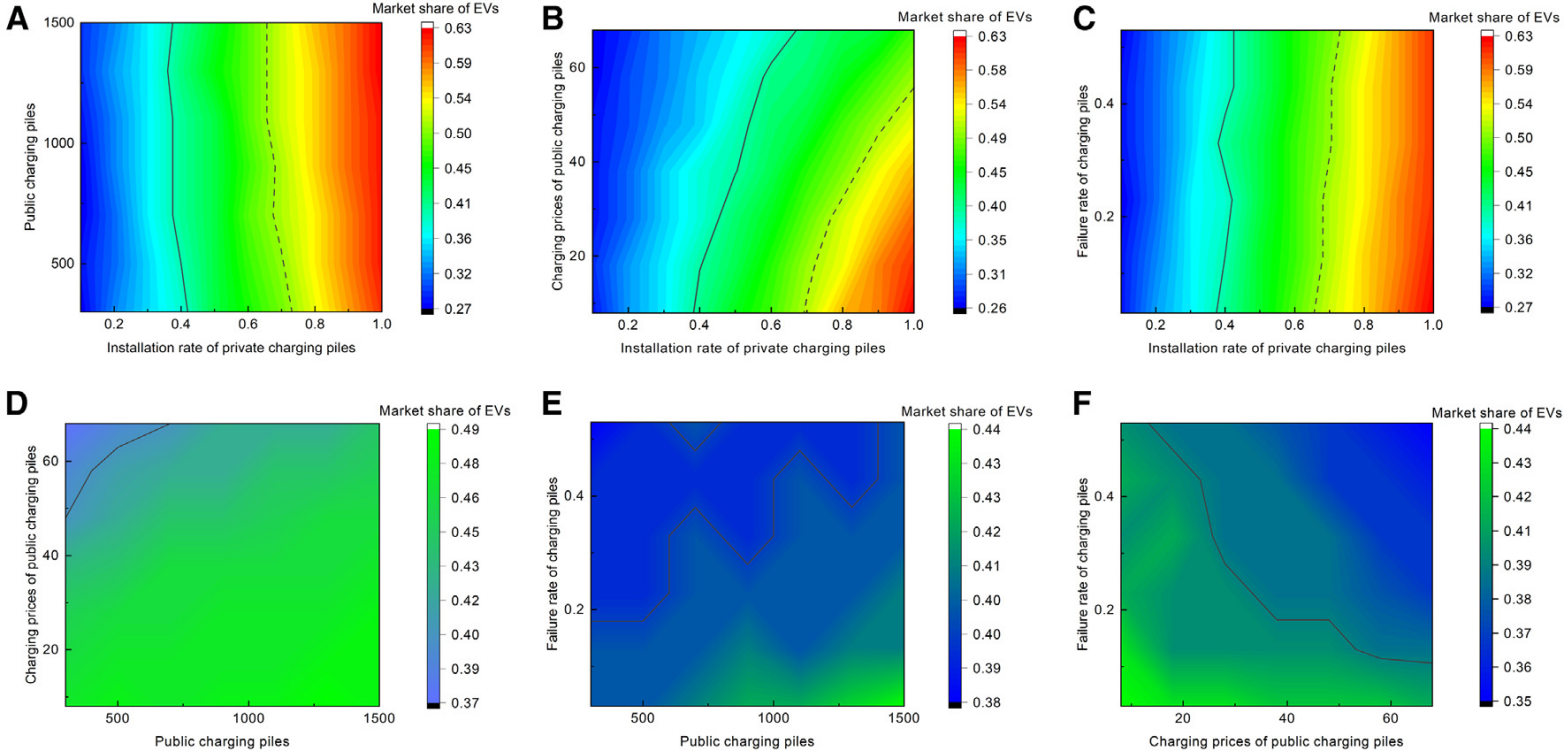
Market share of EVs in steady state under combinations of factors (A) Shows the market share of EVs under varying combinations of the number of public charging piles and the installation rate of private charging piles. (B) Shows the market share of EVs under varying combinations of the charging prices of public charging piles and the installation rate of private charging piles. (C) Shows the market share of EVs under varying combinations of the failure rate of charging piles and the installation rate of private charging piles. (D) Shows the market share of EVs under varying combinations of the charging prices of public charging piles and the number of public charging piles. (E) Shows the market share of EVs under varying combinations of the failure rate of charging piles and the number of public charging piles. (F) Shows the market share of EVs under varying combinations of the failure rate of charging piles and the charging prices of public charging piles. The black line represents the combination of the market share of EVs at 0.39, i.e., the market share of EVs in a steady state under the baseline scenario. The black dotted line indicates the combination at 0.5 market share.

To further analyze how various charging infrastructure factors influence EV market share, market simulations were conducted under different factor combinations, and the resultant market share under stable conditions was analyzed. Figure 6 shows the market share of EVs under different combinations, with the black line combination representing the boundary to maintain the current development trend. It found that the market share of EVs will be at a low level under the conditions of high charging prices, high failure rates, and low installation rates of private charging piles. And in these cases, improving other factors will hardly help to increase the market share of EVs. When the market share of EVs is greater than 0.5 (above the black dashed line threshold), it is considered that EVs have become mainstream vehicles. It found that these combinations are characterized by a high installation rate of private charging piles, while having an appropriate number of public charging piles, a low failure rate, and a low charging price. Therefore, to increase the market share of EVs, the priority is to ensure stable charging prices and low failure rates, and then increase the installation rate of private charging piles and the number of public charging piles.

### Scenario D analysis: Policy intervention in charging facility construction

#### Scenario D-1: Changes in charging pile subsidy rate

**Figure 7:**
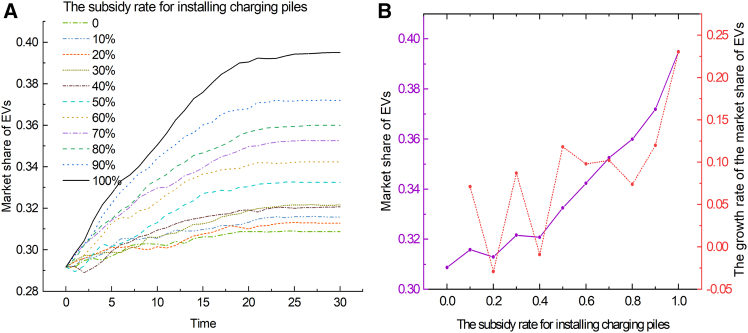
The impact of the change of charging pile subsidy on the market share of EVs (A) Shows the evolution curves of the market share of EVs over time under varying charging pile subsidy rates. (B) Shows the relationship between the charging pile subsidy rate and the market share of EVs (solid purple line), and the growth rate of the market share of EVs (red dashed line), at Time = 30.

To study the impact of the charging pile subsidy rate on the market share of EVs, a simulation was conducted, and the results are shown in Figure 7. As the subsidy rate increases, the market share of EVs shows a gradual upward trend, because higher subsidy rates can reduce the cost of using EVs and enhance consumers’ purchasing motivation. At lower subsidy rates, the market share of EVs is relatively low and fluctuates greatly, which may reflect consumers’ concerns about excessive costs or imperfect charging infrastructure. In addition, consumers’ sensitivity to subsidy rates is a dynamic process. During the initial phase (first 20 ticks), when the subsidy rate was high (50%–100%), the market share of EVs exhibited accelerated growth. As subsidy rates gradually decreased to 0–40%, the growth rate of market share decelerated, eventually stabilizing or showing a slight decline. This trend reflects that consumers are more sensitive to changes in subsidy rates in the early stages of the market. When the subsidy rate is high, consumers are more sensitive to the cost advantages of EVs, have a higher degree of acceptance, and are willing to adopt EVs faster. Reduction in subsidies appears to have dampened consumer purchasing intention. However, in the later phase (beyond 20 ticks), the market share remains relatively stable. It means that consumers’ sensitivity to changes in subsidy rates gradually decreases.

It is worth noting that even under different subsidy rates, the market share of EVs has generally shown a gradual upward trend, indicating that with appropriate policy support, the EV market can grow steadily. The 50% subsidy rate is particularly noteworthy. If combined with the national subsidy reduction policy, the 50% subsidy rate can play a buffering role, promote a smooth transition, and avoid market shocks and a sharp decline in consumer acceptance.

#### Scenario D-2: Changes in private charging pile sharing policy

**Figure 8:**
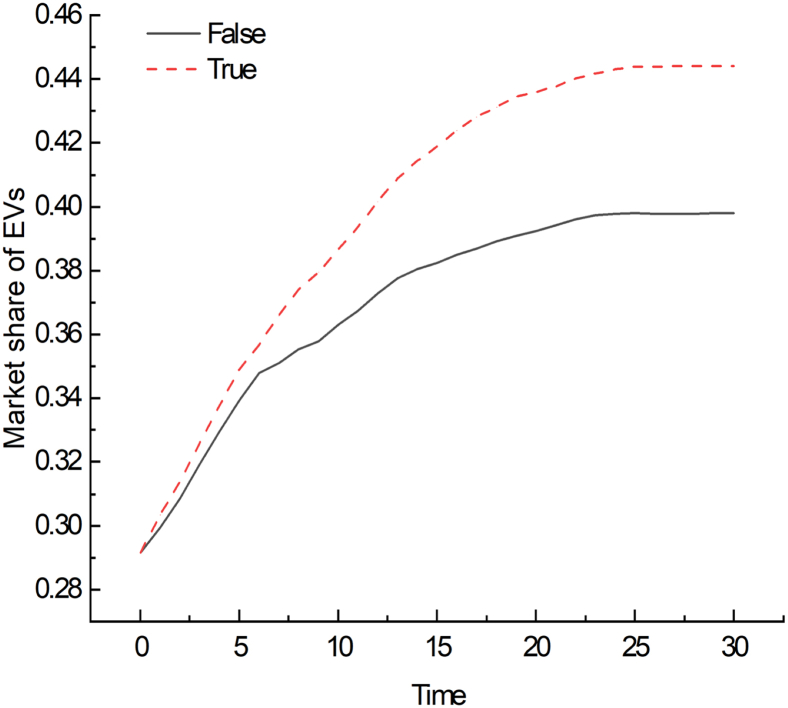
The impact of the private charging pile sharing policy on the market share of EVs

The simulation results of the private charging pile sharing policy are shown in Figure 8. After the implementation of the charging pile sharing policy, the market share is higher than in the scenario without the policy. In the steady state, the market share increased by 13%. It shows that the sharing policy can promote the development of the EV market. However, the initial growth rate of the EV market share is fast, and then gradually stabilizes. This reflects that consumers are more susceptible to the policy in the initial stage, and the later growth rate may be limited by factors like market saturation.

### Conclusions

To study the impact of various factors related to charging infrastructure on the EV market, a multi-agent model of EV-CIC is established based on CAS. The key influencing factors are first identified involving the number of public charging piles, the installation rate of private charging piles, the failure rate of charging piles, the charging electricity price of public charging piles, as well as the subsidy rate of charging piles and the sharing policy of private charging piles. Different scenarios were simulated considering the key factors and their combinations. Several important conclusions, which are briefly summed up, including further policy implications, are described as follows.

(1) The number of charging piles and the installation rate of private charging piles are positively correlated with the EV market share, while the failure rate of charging piles and charging price are negatively correlated with the EV market share. Moreover, analysis reveals that the installation rate of private charging piles has the most significant impact on the market share, followed by the number of public charging piles, the failure rate of charging piles, and finally the charging price of public charging piles. Based on these impact magnitudes, the following adjustment order is recommended: First, increase the installation rate of private charging piles. Second, expand the coverage of public charging piles. Third, reduce the failure rate of charging piles to enhance the reliability and stability of the facilities. Finally, although the charging price of public charging piles shows a relatively lower impact, maintaining the stability of the charging price is essential for ensuring sustained EV market growth and long-term development.
(2) Under conditions characterized by high charging prices, high failure rates, and low private charging pile installation rates, EV market share remains persistently low. In such adverse scenarios, improvements in other factors yield minimal impact on market share enhancement. Analysis reveals that combinations achieving high EV market penetration consistently feature high private charging pile installation rates, adequate public charging infrastructure, and a low failure rate. Therefore, to increase the market share of EVs, the first thing is to ensure stable charging prices and low failure rates, and on this basis increase the installation rate of private charging piles and the number of public charging piles. Notably, the installation of private charging piles may bring breakthroughs to the development of the EV market, and policymakers and stakeholders should pay more attention to this.
(3) Charging pile subsidies and private charging pile sharing policies are important measures to promote the development of the EV market. The effect of sharing policies is more significant than that of increasing subsidy rates. High subsidy rates can be an effective strategy to rapidly expand the EV market, especially in the early stages of promotion. However, as the market matures, the influence of subsidy rates on consumers gradually weakens. This is consistent with the results of Yang et al., who found that the existence of charging pile subsidies increased consumer satisfaction, but the effect was limited.^49^ In contrast, consumers are more concerned about the convenience of charging and are even willing to pay more for charging services or infrastructure.^50^ Therefore, the implementation of charging pile sharing policies can enhance the market’s ability to continue to grow and lay the foundation for the sustainable development of the EV market. However, this does not mean that charging pile subsidies should be directly canceled to meet the requirements of subsidy reduction, because this may have an adverse impact on the charging infrastructure industry and lead to a decline in the market share of EVs. Instead, a gradual subsidy reduction approach serves as an effective buffer, facilitating the market’s transition toward a sustainable growth model.

### Limitations of the study

Some restrictions still exist due to the complexity of practical problems. First, the scope of research objects is limited. Only pure EVs are considered in the EV-CIC model, excluding plug-in hybrid electric vehicles (PHEVs), which hold a certain share in the automotive market. And the field of private passenger cars is the focus of the market acceptance of EVs, without considering the public domain. In fact, the diffusion of EVs in the public domain played a vital role in the early development of EVs. Furthermore, the survey data are predominantly collected from urban commuters, with limited representation of users who frequently engage in intercity travel. This sampling bias may underestimate the importance of fast charging piles in the model. Second, the model construction is simplified. In the EV-CIC model, the heterogeneity and transportation differences between cities have not been taken into account. Meanwhile, the modeling of the consumer decision-making process is simplified, where complex influencing factors in the decision process are not comprehensively addressed. The competitive relationships among charging facility suppliers are not incorporated into consideration, which may compromise the model’s accuracy in predicting market dynamics. The evaluation of policy effectiveness remains largely static, with the absence of a dynamic analytical framework. Third, the scenario settings are restricted. In our model, consumers’ charging demands are primarily assumed to arise from daily commuting. Since fast charging demands are mainly associated with intercity travel scenarios—which are excluded from the consumer decision-making elements—the ratio of fast to slow charging piles demonstrates limited influence in our model. Meanwhile, various dynamic factors are not sufficiently incorporated in the EV-CIC model, including population migration patterns, the diversity of charging facility operation modes, and power grid capacity constraints, the absence of which may compromise the accuracy of model predictions.

In the future, model enhancements can be pursued in several key aspects: incorporating PHEVs and expanding the survey sample coverage; integrating heterogeneity and traffic differences between cities to strengthen the characterization of urban distinctiveness; augmenting the consideration of consumer individual heterogeneity to refine the consumer decision-making process modeling; and differentiating the impact mechanisms of fast and slow charging by incorporating intercity travel scenarios. Furthermore, the robustness of the model is enhanced through verification and optimization with a large amount of real data, thereby improving its utility and predictive accuracy, providing more reliable decision-making support for EV market development and charging infrastructure planning.

## Resource availability

### Lead contact

Further information and requests for resources should be directed to and will be fulfilled by the lead contact, Donghui Liu (mary_ldh16@163.com).

### Materials availability

This study did not generate new unique reagents.

### Data and code availability

• Data: this paper analyzes existing, publicly available data that are listed in the key resources table.
• Code: this paper does not report the original code.
• All other items: any additional information required to reanalyze the data reported in this paper is available from the lead contact upon request.

## Acknowledgments

This work was supported by the 10.13039/501100001809National Natural Science Foundation of China (No.42101300), the Basic Science Center Project of the 10.13039/501100001809National Natural Science Foundation of China (No.72088101, the Theory and Application of Resource and Environment Management in the Digital Economy Era), the Humanities and Social Science, Research Project of Hebei Education Department (SQ2022087), and the 10.13039/501100012226Fundamental Research Funds for the Central Universities (3-7-8-2023-01).

## Author contributions

Conceptualization, D.L., X.G., and N.J.; methodology, D.L. and Y.Z.; software, Y.Z.; formal analysis, J.W.; data curation, Y.Z. and D.L.; writing—original draft, Y.Z. and D.L.; writing—review and editing, D.L., Y.Z., and J.W.; visualization, Y.Z. and D.L.; supervision, D.L., F.A., and X.G.; project administration, D.L. and F.A.; funding acquisition, F.A., D.L., X.G., and N.J.

## Declaration of interests

The authors declare no competing interests.

## STAR★Methods

### Key resources table

**Table:** 

REAGENT or RESOURCE	SOURCE	IDENTIFIER
**Deposited data**		

Consumers’ scores on various indicators, see Table S1	This paper	N/A
Consumers' purchasing intention, see Table S2	This paper	N/A
Area of cities in Hebei Province, see Table S3	Hebei Statistical Yearbook	http://tjj.hebei.gov.cn/hetj/tjnj/2023/zk/indexch.htm
Number of charging stations and gas stations in cities in Hebei Province, see Table S3	AMap Open Platform	https://lbs.amap.com/
Experts’ scoring information, see Tables S4 and S5	This paper	N/A

**Software and algorithms**		

NetLogo 6.3.0	Center for Connected Learning and Computer-Based Modeling, Northwestern University, Evanston, IL.	https://ccl.northwestern.edu/netlogo/
SPSS 25.0	SPSS Statistics Software: Version 25. Armonk, NY: IBM Corp.	https://www.ibm.com/products/spss-statistics
Origin 2021	OriginLab Corporation, Northampton, MA.	https://www.originlab.com/origin

### Experimental model and study participant details

This study employs a multi-agent modeling approach to investigate the impact of charging infrastructure on electric vehicle market diffusion. The consumer samples were drawn from residents of Hebei Province, China. All survey participants signed informed consent forms, and all data were anonymized to protect participant privacy.

### Method details

#### Theory based and model framework

To study the impact of charging infrastructure on the diffusion of EVs, a multi-agent model of electric vehicles-charging infrastructure construction (EV-CIC) is constructed. The EV-CIC system comprises three major subsystems: the consumer decision-making system, the automobile manufacturer system, and the charging facility supplier system. These subsystems are interconnected through complex interaction relationships through the behaviors of agents. Figure S1 shows the framework of the EV-CIC system, which involves key agents consisting of consumers, automakers, government, and charging facility suppliers, and the interactive mechanism between agents, which is based on the theory of market behavior. The decisions of each agent are primarily determined by their attributes, interactions among agents, and interactions between agents and the environment, in turn, influencing the behavior of agents, which creates a complex adaptive system. Consumer behavior is the driving force in the EV market.^51^ The purchasing decisions of consumers are determined by their attributes, the interaction between consumers and automakers, charging pile suppliers, and the government, and the interaction between consumers and consumers.^3^^,^^46^ Consumer preferences are determined by their attributes, such as whether they have any car, current vehicle age, and assigned purchase age, and whether they identify as environmentalists or innovators. The factors related to vehicles mainly include their economy and performance, which are determined by automakers. These automakers adjust production costs using technology learning curves and then set sales prices through a cost-plus pricing strategy.^29^ The production costs and selling prices fluctuate with the market demand for EVs, and the changes in the prices further influence consumer purchasing intentions. The coverage and quality of charging facilities impact consumer acceptance of EVs, thereby affecting the diffusion of EVs.^52^ Charging facility suppliers plan their infrastructure based on the installation rate of EVs and government policies, which in turn further influences consumer purchasing intentions. Government agents, acting as market regulators, implement economic policies (such as purchase incentives and subsidies for charging piles) and social policies (such as vehicle restriction measures and charging pile sharing policy), shaping the automotive market environment and influencing the behavior of other agents.^53^^,^^54^ Additionally, real-world social networks exhibit characteristics of small-world networks; therefore, this study utilizes the small-world network model as the basis for analyzing consumer social networks.^55^ The model is beneficial for gaining a more comprehensive understanding of how various factors related to charging piles dynamically impact the EV market.

Based on the EV-CIC system framework, the model was conducted in the NetLogo simulation environment. We developed the system's operational processes to further complement the details, demonstrating the actual workflows and interaction mechanisms. Figure S2 shows the workflow and interaction mechanism of the EV-CIC system. Starting from t=1, the process begins with consumer decision-making. The system evaluates consumer attributes individually, determines their purchase intention, and calculates the relative utility value (U) for consumers with purchase intention. When the U value exceeds 1, the consumer chooses to purchase a battery electric vehicle (BEV); otherwise, they opt for a conventional vehicle (CV). After each consumer decision, the system immediately updates that consumer's attributes and proceeds to evaluate the next consumer until all consumer decisions for that period are completed. Once all consumer decisions in a simulation period are finalized, the system conducts market statistics, tracking and recording the sales data of BEVs and CVs, monitoring the total volume of both vehicle types, and calculating the market share of electric vehicles. These market data directly influence the decision-making behavior of charging facility suppliers, prompting them to update private charging infrastructure and adjust public charging infrastructure accordingly. Meanwhile, automakers also update vehicle production costs and sales prices based on market conditions. The agent is updated asynchronously in every simulated period, and the simulation process continues until the completion of 30 periods.

To ensure that the model operates within a reasonable context and generates meaningful results, fundamental assumptions have been established. These assumptions provide the necessary framework and boundary conditions for the construction and simulation of the model. Assumptions are as follows: (1) The market development environment is stable, and inflation is not considered; (2) Only pure EVs are considered for new energy vehicles; (3) Consumers will not delay purchases; (4) The social network conforms to the NW small-world structure and remains unchanged throughout the process; (5) The adjustments of government policy have immediate effects without any lag; (6) The performance metrics of charging facility achieve specified standards; (7) There will not be breakthroughs in automobile production technology, and the layout of fuel stations will not change. (8) Consumers’ charging demands primarily stem from intracity travel, with limited consideration given to intercity travel needs.

#### The decisions of multiple agents

##### The decisions of consumer purchase

The decisions of consumer purchase, as shown in Figure S3, are key to EV-CIC. First, four questions were used to pick out consumers. Next, the relative utility value of EVs relative to traditional vehicles is calculated through the relative utility function. Finally, a purchase decision is made based on the result of the relative utility calculation.

A multi-dimensional relative utility function is constructed, that integrates vehicle utility, charging facility utility, policy utility, and social utility through a weighted approach. The relative vehicle utility primarily evaluates the comprehensive advantages of electric vehicles compared to conventional vehicles, including economic and performance characteristics. The relative charging facility utility reflects the service level of charging infrastructure, encompassing factors such as convenience, reliability, economic efficiency, intelligence level, and environmental friendliness. The relative policy utility quantifies the impact of government support policies, including the assessment of policy measures such as purchase subsidies and usage rights. The relative social utility, based on small-world network theory, reflects the demonstration effect in social networks, representing the influence of the surrounding environment on individual decision-making.^55^ If the relative utility U value is greater than 1, that indicates the BEVs have an advantage, and consumers will choose to purchase BEVs; otherwise, consumers will choose to purchase CVs.

Due to the heterogeneity of consumer preferences for different factors,^25^^,^^56^ each consumer scores the indicators (since economic policies impact vehicle and charging facility economics, scoring is only applied to social policies, not economic ones). Based on the scores, the weight of each indicator is calculated and remains unchanged. The relative utility is calculated by Equation 1.

(Equation 1) $$U=w_{v}*v_{t}+w_{e}*e_{t}+w_{p}*p_{t}+w_{s}*s_{t}$$

where $v_{t},e_{t},p_{t},s_{t}$ represent the relative vehicle utility, relative charging facility utility, relative policy utility, and relative social utility. $w_{i}$ denotes the weight of i, and $\sum w_{i}=1\left(i=v,e,p,s\right)$.

(1) The relative vehicle utility $v_{t}$ is composed of the car's relative economic utility $v_{t1}$ and relative performance utility $v_{2t}$,^45^^,^^56^^,^^57^ calculated by Equation 2.

(Equation 2) $$v_{t}=wt_{vt1}*v_{t1}+wt_{vt2}*v_{t2}$$

where $v_{t}$ represents the total vehicle utility value, $wt_{vt1}$ represents the weight coefficient of economic utility, $v_{t1}$ represents the economic utility value, $wt_{vt2}$ represents the weight coefficient of performance utility, and $v_{t2}$ represents the performance utility value.

The economic utility is primarily calculated by comparing the Annual Cost of Ownership (ACO) between electric vehicles and conventional vehicles.^58^^,^^59^ This function considers purchase cost, maintenance cost, related taxes, and residual value, while also accounting for EV-specific factors such as battery replacement costs and government subsidies.^58^^,^^59^ Given the current characteristics of EV battery life, the battery replacement cycle is set to 5-year intervals.^60^ The relative economic utility is calculated by Equation 3.

(Equation 3) $$v_{t1}=\frac{\frac{P_{2}+C_{2}+F_{2}-P_{2}*R_{2}}{Y_{2}}}{\frac{P_{1}+C_{1}+F_{1}+C_{b}*ROUND\left(Y_{1}/5\right)-P_{1}*R_{1}-S_{1}}{Y_{1}}}$$

where P_i_ represents the purchase price of electric and conventional vehicles, $C_{i}$ represents maintenance costs, F_i_ represents related taxes, R_i_ represents residual rate, Y_i_ represents expected service life. When i=1, it represents electric vehicles; when i=2, it represents conventional vehicles. $C_{b}$ represents battery replacement cost, S_1_ represents government subsidies for electric vehicles, and $ROUND\left(Y_{1}/5\right)$ represents the number of battery replacements (once every 5 years).

The performance utility $v_{t2}$ employs a comprehensive evaluation method based on five key performance indicators, including safety, technological advancement, energy consumption level, noise level, and carbon emissions.^16^^,^^39^^,^^46^^,^^47^ A square root normalization method is applied to effectively mitigate the impact of extreme values on evaluation results, ensuring more objective and fair assessments.^16^ And this normalization approach is consistently applied throughout the subsequent Equations. The relative performance utility, calculated by Equation 4, is obtained based on expert scoring of representative car features.

(Equation 4) $$v_{t2}=\frac{\sum_{j=1}^{5}\sqrt{\frac{score-v_{1j}}{score-v_{2j}}}}{5}$$

where $score-v_{ij}$ represents the scores, and the indices from j=1 to j=5 represent the following five performance attributes of a vehicle: safety, technological level, energy consumption level, low noise, and carbon emissions.

(2) The utility of charging facilities is quantified through a weighted summation of five fundamental aspects: convenience, reliability, economic efficiency, intelligent connectivity, and environmental sustainability. The convenience assessment primarily evaluates charging duration and facility accessibility.^16^^,^^17^ The reliability evaluation framework incorporates three essential indicators derived from equipment management theory: failure rate, maintenance time, and service life.^61^ The economic assessment methodology employs annual average cost analysis, encompassing charging costs, equipment investment, and operational maintenance considerations.^39^^,^^45^ The intelligent connectivity evaluation is grounded in intelligent transportation system assessment theory, examining smart management capabilities, information interaction efficiency, and user experience metrics.^47^ The environmental sustainability assessment focuses on energy consumption rates and resource utilization efficiency.^62^^,^^63^ The calculation method for evaluating the utility of the relative charging facility $e_{t}$ is calculated by Equation 5.

(Equation 5) $$e_{t}=\sum_{i=1}^{5}wt_{e_{ti}}*e_{ti}$$

where $e_{t1}$ to $e_{t5}$ represent consumers' evaluation of charging facility in terms of convenience, reliability, economics, intelligent networking, and eco-friendliness, calculated by Equations 6 to 10 respectively. wt represents the weight coefficient of each evaluation indicator.

(Equation 6) $$e_{t1}=\frac{\left(\sqrt{\frac{t_{2}}{\sigma_{1}*t_{11}+\sigma_{2}*t_{12}}}+\sqrt{\frac{n_{11}*\left(1-tr_{1}\right)+p_{s}*n_{p-piles}}{n_{21}}}\right)}{2}$$

where $t_{2}$ represents the time required for refueling; $t_{11}$ and $t_{12}$ denote the time needed for fast and slow charging, respectively; $\sigma_{1}$ and $\sigma_{2}$ represent the proportions of utilizing fast and slow charging; $n_{11}$ indicates the number of public charging piles within 10 patches; $tr_{1}$ is the failure rate of public charging piles; $p_{s}$ denotes the private charging pile sharing policy (1 if implemented, 0 otherwise); $n_{p-piles}$ represents the number of private charging piles within 10 patches; and $n_{21}$ is the number of gas stations within 10 patches. The calculation of convenience utility takes into account the spatial distribution of infrastructure in different cities.

(Equation 7) $$e_{t2}=\frac{\sqrt{\frac{tr_{2}}{tr_{1}}}+\sqrt{\frac{re_{2}}{re_{1}}}+\sqrt{\frac{l_{1}}{l_{2}}}}{3}$$

where $tr_{1}$ and $tr_{2}$ represent the failure rates of public charging piles and gas stations; $re_{1}$ and $re_{2}$ denote their average maintenance times; $l_{1}$ and $l_{2}$ indicate their average service life spans.

(Equation 8) $$e_{t3}=\frac{p_{2}*trip*365}{\left(\left(\lambda_{1}*p_{11}+\lambda_{2}*p_{12}\right)*trip*Y_{1}*365+C_{ip}\right)/Y_{1}}$$

where $p_{2}$ represents the fuel price per hundred kilometers; trip denotes the planned daily mileage; $p_{11}$ and $p_{12}$ represent the charging cost per hundred kilometers for public and private charging piles; $\lambda_{1}$ and $\lambda_{2}$ represent the proportions of consumers using public and private charging piles; $Y_{1}$ indicates the expected service life of electric vehicles; $C_{ip}$ represents the installation cost of private charging piles borne by consumers.

(Equation 9) $$e_{t4}=\frac{\sum_{k=1}^{3}\sqrt{\frac{score-e_{1k}}{score-e_{2k}}}}{3}$$

where $score-e_{ik}$ represents the evaluation scores, and the indices from k=1 to k=3 represent three performance attributes: Intelligent management level, Information interaction capability, and User experience.

(Equation 10) $$e_{t5}=\frac{\sqrt{\frac{cs_{1}}{cs_{2}}}+\sqrt{\frac{use_{1}}{use_{2}}}}{2}$$

where $cs_{1}$ and $cs_{2}$ represent the energy consumption rates of electric and conventional vehicles; $use_{1}$ and $use_{2}$ represent the resource utilization rates of electric and conventional vehicles.

(3) The relative policy utility, calculated by Equation 11, primarily focused on social policies:

(Equation 11) $$p_{t}=wt_{pn}*p_{n}+wt_{ps}*p_{s}$$

where $p_{n},p_{s}$ represent the license plate restriction policy and charging pile sharing policy. If the policy is open, it is 1, otherwise it is 0.

(4) Watts and Strogatz pointed out that interpersonal networks in the real world have the characteristics of small-world networks, which are considered to be the most influential and most commonly used networks.^30^ Therefore, this study also uses small-world networks as the basis of consumer interaction networks. The relative social utility evaluation is calculated by Equation 12.

(Equation 12) $$s_{t}=\frac{n_{bev}}{n_{cv}}$$

where $s_{t}$ represents the social network utility score; $n_{bev}$ denotes the number of electric vehicles owned by connected individuals within the social network; $n_{cv}$ indicates the number of conventional vehicles owned by connected individuals within the social network.

##### The behavior of automakers

Automakers are highly sensitive to market demand, with production costs adjusted through technological learning, which influences vehicle prices and subsequently affects consumer decisions. Production cost changes are modeled using a technological learning curve as Equation 13, while vehicle prices are determined through cost-plus pricing as Equation 14.

(Equation 13) $$C\left(Q\right)=C_{0}*Q^{-\alpha }$$

where C(Q) is the unit vehicle production cost when the vehicle output reaches Q, $C_{0}$ is the initial production cost, and α is the technological advancement speed of the automaker.

(Equation 14) $$P_{i}=C_{i}*\left(1+\beta \right)$$

where $P_{i}$ represents the sales price of product i, and β represents the profit margin of the product.

##### The behavior of charging facility suppliers

Charging facility suppliers are responsible for planning the number of charging piles and setting charging prices, playing a vital role in the development of the electric vehicle market. Suppliers need to reasonably determine the number of charging piles based on factors such as the number of electric vehicles, regional demand, and installation conditions. At the same time, they must establish reasonable pricing standards by considering electricity pricing policies and market structures to meet market demand and achieve long-term profitability. For the public charging pile market and the private charging pile market, the behavior of suppliers shows different characteristics and patterns.

(1) The behavior in the public charging pile market

The planning of public piles is determined by the current electric vehicle ownership and the vehicle-to-charging pile ratio. Specifically, the required number of public charging piles is calculated by dividing the total number of electric vehicles by the target vehicle-to-pile ratio, as Equation 15. The total number is then allocated between fast and slow charging in a 3:2 ratio.

(Equation 15) $$N_{pub}\left(t\right)=\frac{N_{ev}\left(t-1\right)}{R_{tt}}$$

where $N_{pub}\left(t\right)$ represents the number of public charging piles at time t; $N_{ev}\left(t-1\right)$ denotes the electric vehicle ownership at time t-1; $R_{tt}$ indicates the target vehicle-to-pile ratio.

Given the significant regional disparities in charging pile distribution, this model maintains each area's historical proportion of the provincial charging infrastructure, as Equation 16. These proportions are derived from Hebei Province's regional public charging pile distribution data in 2023. Then the number of charging piles in each city is calculated by Equation 17.

(Equation 16) $$w_{city}=\frac{N_{pub,city}\left(t_{0}\right)}{\sum_{k=1}^{n}N_{pub,k}\left(t_{0}\right)}$$

(Equation 17) $$N_{pub,city}\left(t\right)=w_{city}*N_{pub}\left(t\right)$$

where $w_{city}$ represents the distribution weight coefficient for region city; $N_{pub,city}\left(t_{0}\right)$ denotes the number of public charging piles in region city at baseline time t_0_; $\sum_{k=1}^{n}N_{pub,k}\left(t_{0}\right)$ represents the total number of public charging piles in the province at time t_0_, with n=11 administrative regions in Hebei Province; $N_{pub,city}\left(t\right)$ indicates the number of public charging piles in region city at time t.

In addition, the charging price of public charging piles is affected by regional grid pricing, peak-valley periods, and supplier market structure. It is mainly composed of the basic electricity price set by the government and the service fee set by the charging facility supplier. It is integrated into a comprehensive variable $P_{11}$ in the model to observe its impact on the EV market for the convenience of model operation. As for the installation cost of public charging piles, suppliers recover their investment through long-term service fees rather than directly charging installation fees, thus this factor is not considered in the impact analysis of this model.

(2) The behavior in the private charging pile market

The planning of private piles primarily depends on new electric vehicle acquisitions and installation conditions. Not all electric vehicle purchasers have suitable conditions for installing private charging piles, as this is influenced by various factors such as the availability of dedicated parking spaces and building installation requirements. Therefore, we introduce an installation feasibility coefficient κ. The number of private charging piles in each period equals the previous period's quantity plus new installations, with all private charging piles designated as slow-charging units, calculated by Equation 18.

(Equation 18) $$N_{pri}\left(t+1\right)=N_{pri}\left(t\right)+\kappa *\Delta N_{ev}\left(t\right)$$

where $N_{pri}\left(t+1\right)$ represents the number of private charging piles at time t+1; $N_{pri}\left(t\right)$ denotes the quantity at time t; κ is the installation feasibility coefficient; $\Delta N_{ev}\left(t\right)$ indicates the number of new EVs at time t, that is, the number of consumers who purchased EVs at time t.

In addition, the charging price of private charging piles primarily follows the government-guided tiered electricity pricing for residential use, with the model adopting an average value $P_{12}$ as the charging price. The total cost of installing a private charging pile encompasses hardware equipment costs, installation fees, and supporting facility costs, as Equation 19. The actual installation cost borne by consumers equals the total installation cost minus government subsidies, as Equation 20.

(Equation 19) $$fee_{p}=C_{p}+C_{i}+C_{f}$$

(Equation 20) $$C_{ip}=fee_{p}*\left(1-S_{2}\right)$$

where $fee_{p}$ represents the total cost of installing a private charging pile; $C_{p}$ denotes hardware equipment costs; $C_{i}$ represents installation fees; $C_{f}$ indicates supporting facility costs; $C_{ip}$ represents the actual installation cost borne by consumers after subsidies; $S_{2}$ denotes the comprehensive subsidy rate.

##### The behavior of the government

The government plays an important role of regulator and promoter, formulating different policies based on market conditions, primarily including economic policies and social policies. Economic policies include subsidies for electric vehicles S_1_ and the subsidy rate for charging piles S_2._ Social policies include license plate restriction policies and charging pile sharing policies. When the market's EV ownership reaches the expected market share, the government considers the development of EVs to be relatively improved and adjusts the policies accordingly.

#### Parameter settings and model testing

The EV-CIC was conducted in the NetLogo simulation environment, which is based on Hebei Province. The EV-CIC model is a logical system model designed to explore the interactive relationships and influence mechanisms among multiple subjects in the electric vehicle market. Focusing on a specific region enables more targeted and accurate analysis, despite significant variations in charging infrastructure development across different regions. Hebei Province, as a province with a medium level of economic development in China, is representative. Its research conclusions are more universal and can provide a useful reference for similar regions. Meanwhile, the comprehensive availability of relevant data in Hebei Province provides necessary data support for model construction and validation. Given the significant differences in geographical location, demographic characteristics, charging infrastructure network layout, and vehicle ownership among the 11 prefecture-level cities in Hebei Province, the simulation environment is divided into 11 regions, each defining different agents and their attributes to ensure the model accurately reflects the actual conditions of each area. The model is scaled at a ratio of 1:1000, effectively simulating the vehicle purchase behavior of 546,000 residents in a virtual area.

To ensure the model operates effectively, three datasets are required to map the real data of Hebei Province into the model. The first dataset is obtained through survey sampling, capturing consumer personal attributes. This survey aims to measure the impact of various factors on the willingness to purchase EVs. The questionnaire includes 43 questions divided into 9 dimensions, with 1-8 sub-questions under each dimension. All questions are measured using a Likert scale (1 = Very Unimportant, 2 = Unimportant, 3 = Neutral, 4 = Important, 5 = Very Important). The survey was conducted from January to March 2024, using a combination of online and offline methods, resulting in 415 valid questionnaires. Statistical data were provided on consumer attributes and evaluations of factors such as policies, vehicle characteristics, charging infrastructure, and social networks, which were used to determine the weight of each indicator in consumer decision-making. Consumer scores and purchase intentions are detailed in Tables S1 and S2. The second dataset comes from the Bureau of Statistics, collecting data on Hebei Province's geographical location, population characteristics, and charging infrastructure network layout, as seen in Table S3. The initial ratio of fast charging to slow charging piles (3:2) was set based on the actual distribution of charging facilities in China. The third dataset is derived from expert ratings on vehicle performance and the intelligence and connectivity levels of charging facilities, as shown in Tables S4 and S5.

The effectiveness of the EV-CIC model was systematically verified through a combination of data calibration and logical validation. For data calibration, simulation results from the baseline scenario were compared with historical data from Hebei Province's electric vehicle market. It is evident that the historical data and the modeled data for EV sales exhibit a high degree of consistency over a three-year period, as shown in Figure S4, demonstrating the model's ability to accurately capture real-world market dynamics. For logical validation, a modular validation approach was adopted to ensure that behavioral rules and decision-making processes of various agents align with real-world situations. For example, key parameters such as the number of public charging piles and charging pile failure rates were adjusted to observe changes in electric vehicle market share trends, verifying whether the model's response to parameter adjustments meets logical expectations. The verification results show that the parameter settings of the model meet the logical requirements.

#### Simulation scenarios

To analyze the diffusion of the EV market, Table S6 shows different scenarios, which are simulated and analyzed to examine the effects of various factors. Each scenario is simulated 10 times with 30 ticks, and the average record is taken as the market share of EVs to evaluate the impact of various factors on the market share of EVs.

(1) Scenario A: Baseline scenario

As a baseline scenario, assumptions are primarily based on real-world conditions, and simulations are conducted without changing the initial parameters, to analyze the development trends and potential market size of the EV market. In this scenario, key data settings include: the number of public charging piles at 300, the installation rate of private charging piles at 40%, the failure rate of charging piles at 30%, the charging prices of public charging piles at ¥8, and the charging pile subsidy rate at 100%.

(2) Scenario B: EV diffusions affected by single factors

Scenario B is simulated to evaluate the impact of single factors related to charging infrastructure on the market share of EVs. The single factors include four variables: the number of public charging piles, the installation rate of private charging piles, the failure rate of charging piles, and the charging price of public charging piles. Only one variable is varied in each simulation, while others are at baseline. Specifically, the number of public charging piles is adjusted from 300 to 1500 in increments of 200. The installation rate of private charging piles is adapted progressively from 0% to 100%, with each iteration increasing by 10%. The failure rate of charging piles ranges from 3% to 63%, with a consistent increment of 10% per step. The charging price of public charging piles is altered from ¥8 to ¥68, increasing by ¥10 each time. Subsequently, a detailed analysis is conducted on the impact of each variable on electric vehicle (EV) market share, capturing trends over time and quantifying the marginal effects.

(3) Scenario C: EV diffusions affected by combination factors

Scenario C is simulated to analyze the impact of combination factors on the market share of EVs in a steady state. The pairwise combinations of the four single factors in Scenario 2 can be combined into six resultants. Only two variables from a combination factor are varied in each simulation, while others are at baseline. Subsequently, the boundary levels of combination factors that maintain baseline stability are identified, factors with greater influence are explored, and the effective combinations for promoting the EV market are determined.

(4) Scenario D: EV diffusions affected by policy intervention

Due to the mandatory and authoritative nature of policy, the impact of policy factors on the EV market is studied independently. In this scenario, policy factors include the subsidy rate for charging piles and the private charging pile sharing policy. In the simulation, the subsidy rate is adjusted from 0% to 100%, increasing by 10% each increment. The simulations are aimed at exploring effective measures to promote the development of EVs.

### Quantification and statistical analysis

All statistical analyses of consumer questionnaire data were conducted using IBM SPSS Statistics (Version 25.0). Descriptive statistics were computed for demographic variables, and the results are shown in Tables S1 and S2. The simulation results were generated using NetLogo, followed by analysis and visualization using Origin 2021. The visualization outcomes are shown in Figures 1, 2, 3, 4, 5, 6, 7, 8, and S4.
