# Supplementary material for: Impact of charging infrastructure construction on electric vehicle diffusion based on a multi-agent model
Source: iScience. 2025 Mar 20;28(4):112257. doi: 10.1016/j.isci.2025.112257 (PMC12002657; doi:10.1016/j.isci.2025.112257)
Supplement: Document S1. Figures S1–S4 and Tables S1–S6 [file mmc1.pdf]

iScience, Volume *28*

Supplemental information

Impact of charging infrastructure construction on electric vehicle diffusion based on a multi-agent model

Yingying Zheng, Donghui Liu, Feng An, Jian Wang, Xiangyun Gao, and Nanfei Jia

Supplementary Information

Supplementary Figures:

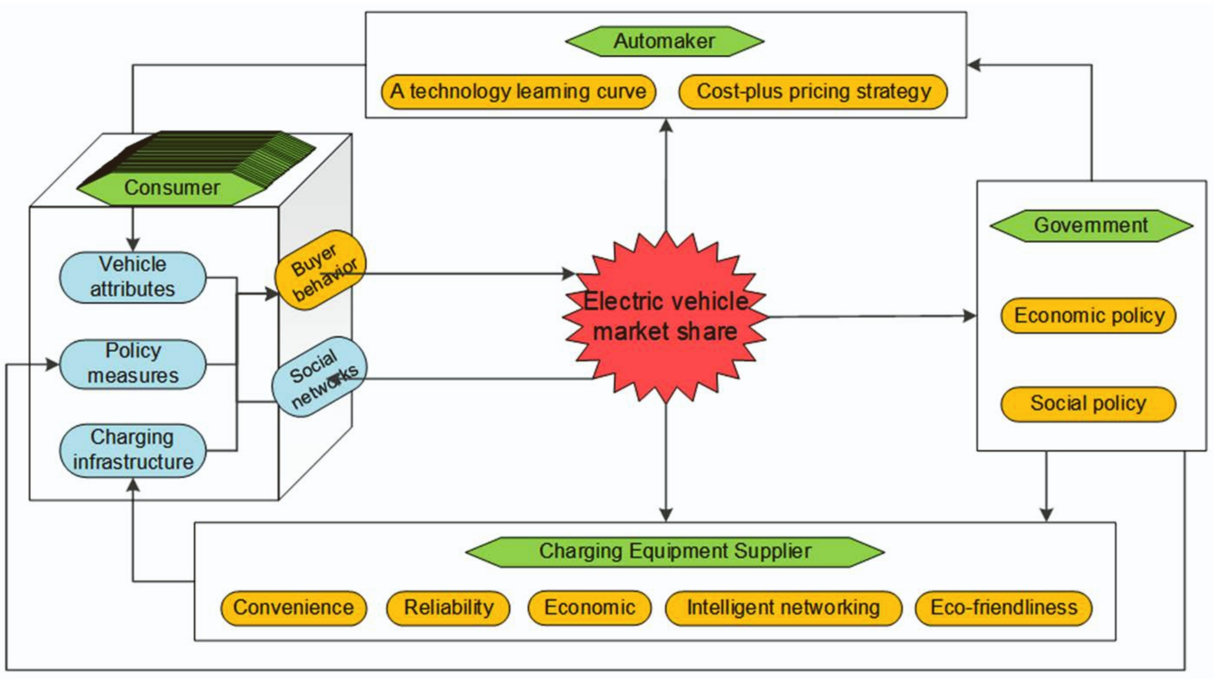

Fig. S1. EV-CIC Framework Diagram based on CAS; Related to STAR Methods. Green diamonds represent agents, orange shapes represent the decision-making behaviors of the agents, and the direction of the arrows indicates the direction of influence (for example, an arrow from 'a' to 'b' indicates that 'a' influences 'b').

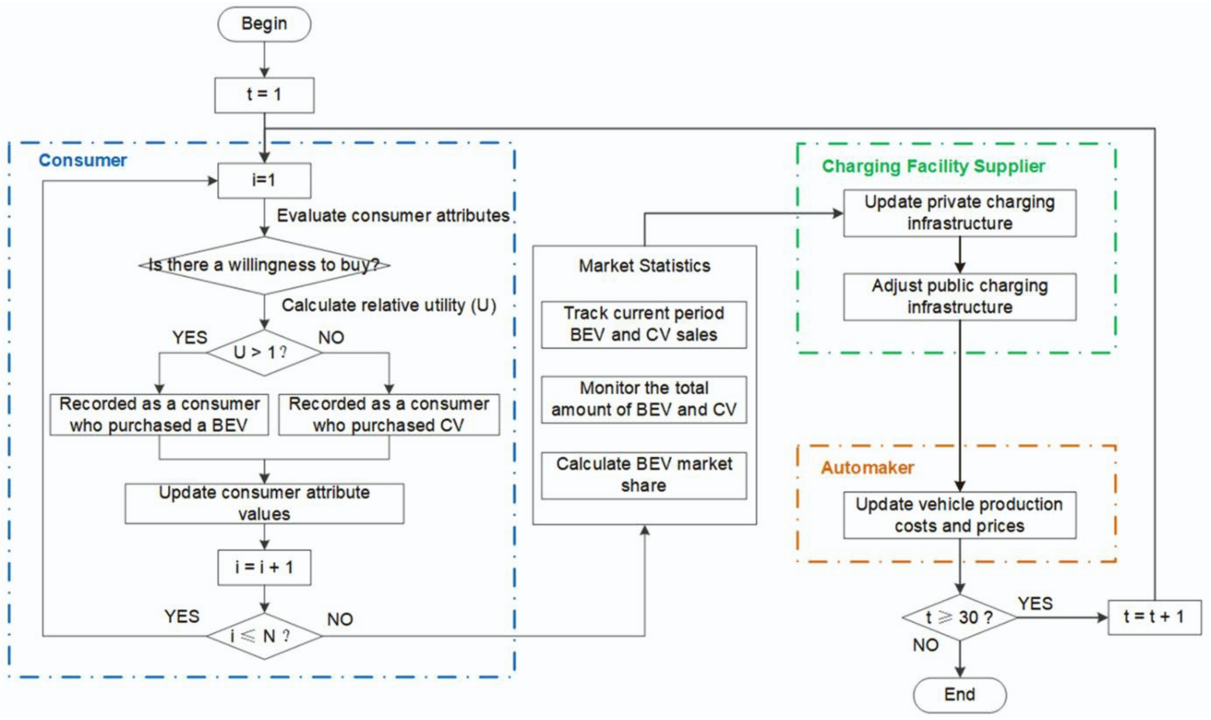

Fig. S2. Simulation flowchart of the EV-CIC model; Related to STAR Methods.

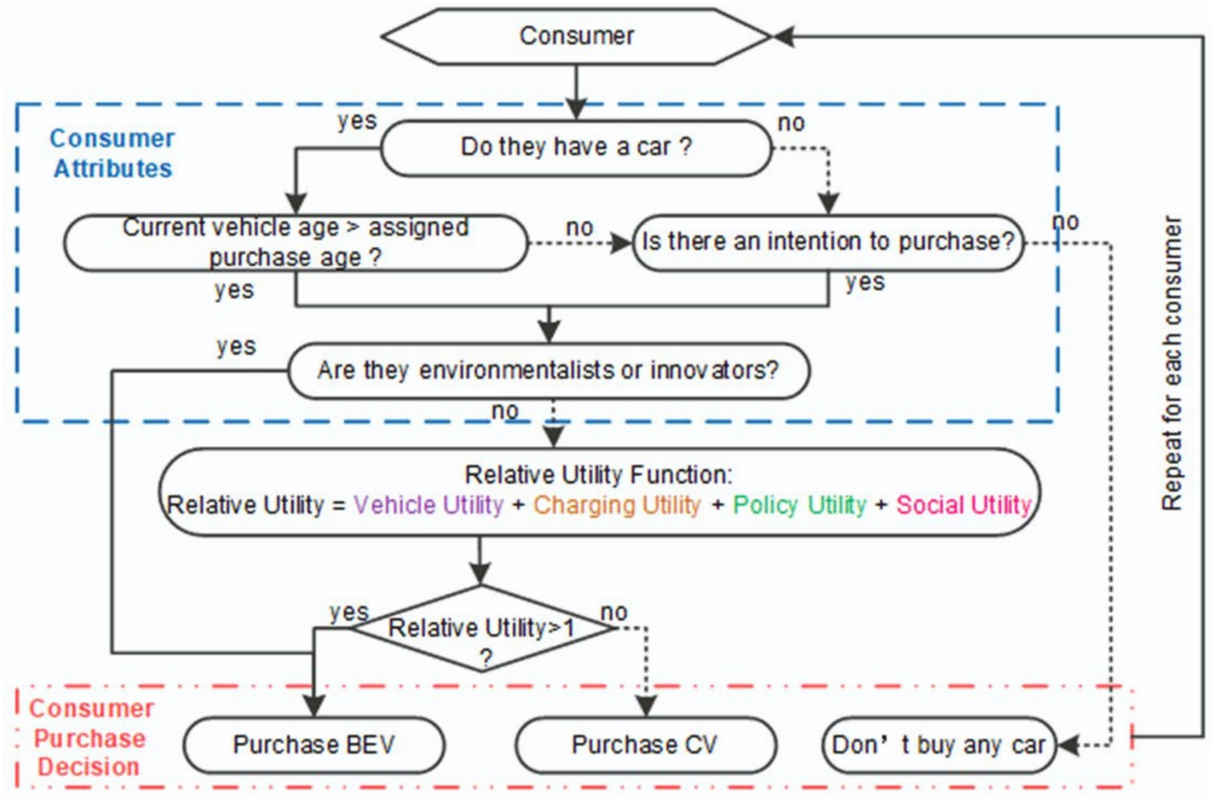

Fig. S3. Consumer purchase decision model; Related to STAR Methods.

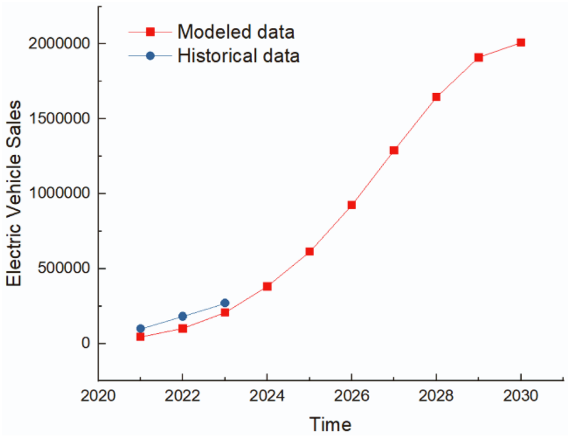

Fig. S4. Comparison of historical data and modeled data for EV sales; Related to STAR Methods.

Supplementary Tables:

Table S1: Consumers' scores on various indicators; Related to Key Resources Table and STAR Methods. Data are presented as percentages of respondents for each importance level. Mean values are calculated on a 5-point Likert scale (1 = Very Unimportant to 5 = Very Important). SD = Standard Deviation.

index	very unimportant	unimportant	neutral	important	very important	Mean ± SD
Social Policy	4%	19%	9%	42%	27%	3.69 ± 1.17
Convenience	0%	35%	17%	34%	14%	3.27 ± 1.09
Reliability	2%	34%	21%	30%	13%	3.16 ± 1.10
Economic	3%	26%	22%	22%	27%	3.45 ± 1.22
Smart and Connected	4%	32%	18%	29%	18%	3.26 ± 1.19
Eco-friendliness	7%	28%	17%	13%	35%	3.41 ± 1.39
Economic Attributes	2%	10%	9%	26%	53%	4.17 ± 1.10
Automobile Performance	4%	12%	7%	46%	32%	3.89 ± 1.09
Social Networks	4%	10%	9%	47%	31%	3.90 ± 1.07

Table S2: Consumers' purchasing intention; Related to Key Resources Table and STAR Methods. Total sample size n=415.

		Do you have the intention to buy a car?	
		Yes	No
Is there a car?	Yes	128	173
	No	85	29

Table S3: Basic data of cities in Hebei Province; Related to Key Resources Table and STAR Methods.

City	Area(km^2)	Number of charging piles	Number of gas stations
Zhangjiakou	36357	307	806
Chengde	39511.89	169	664
Qinhuangdao	7812.4	243	500
Baoding	22135	673	1835
Langfang	6429	759	752
Tangshan	13472	581	1557
Cangzhou	14304.26	326	1155
Shijiazhuang	14530	714	1720
Hengshui	8837	169	723
Handan	12073.8	336	1189
Xingtai	12400	337	1147

Table S4: Experts' scoring information on vehicle performance; Related to Key Resources Table and STAR Methods.

	safety	techinque level	Energy consumption level	low noise	carbon emission
bev	3	5	4	5	5
cv	4	2	3	3	2

Table S5: Experts' scoring information on the intelligent level of charging facility; Related to Key Resources Table and STAR Methods.

	Intelligent management level	Information interaction capability	User experience
charging pile	5	4	4
Gas station	2	2	3

Table S6: Scenario settings for simulation of the EV-CIC model; Related to STAR Methods.

Scenarios	Scenario descriptions	Variables
Scenario A:<br>Baseline scenario	Simulations based on real-world data serve as a basis for other scenario studies.	Stable variables, with no adjustment.
Scenario B:<br>EV diffusions affected by single factors	Simulations are used to analyze the impact of individual infrastructure factors on the EV market, including time trends, overall effects, and marginal effects.	Scenario B-1: changes of the number of public charging piles (n_{i1});<br>Scenario B-2: changes of the installation rate of private charging piles（R_{ip}）;<br>Scenario B-3: changes of the failure rate of charging piles（tr_i）;<br>Scenario B-4: changes of the charging prices of public charging piles（p_{11}）
Scenario C:<br>EV diffusions affected by combination factors	EV diffusions affected by combinations of infrastructure factors in the steady state	Changes of the pairwise combinations of the four single factors in Scenario 2, which can be combined to six resultants
Scenario D:<br>EV diffusions affected by policy intervention	EV diffusions affected by policy intervention including the subsidy rate for charging piles and the private charging pile sharing policy	Scenario D-1: changes of charging pile subsidy rate（S_2）;<br>Scenario D-2: changes of private charging pile sharing policy (p_s)
